# Recent Advances in Multimodal Molecular Imaging of Cancer Mediated by Hybrid Magnetic Nanoparticles

**DOI:** 10.3390/polym13172989

**Published:** 2021-09-03

**Authors:** Yurena Luengo Morato, Karina Ovejero Paredes, Laura Lozano Chamizo, Marzia Marciello, Marco Filice

**Affiliations:** 1 Nanobiotechnology for Life Sciences Lab, Department of Chemistry in Pharmaceutical Sciences, Faculty of Pharmacy, Universidad Complutense de Madrid (UCM), Plaza Ramón y Cajal, 28040 Madrid, Spain; yluengo@ucm.es (Y.L.M.); kovejero@ucm.es (K.O.P.); laurloza@ucm.es (L.L.C.); 2 Microscopy and Dynamic Imaging Unit, Fundación Centro Nacional de Investigaciones Cardiovasculares Carlos III (CNIC F.S.P.), Calle Melchor Fernández Almagro 3, 28029 Madrid, Spain; 3 CIBER de Enfermedades Respiratorias (CIBERES), Melchor Fernández Almagro 3, 28029 Madrid, Spain

**Keywords:** magnetic nanoparticles, multimodal molecular imaging, cancer, MRI, nanobiotechnology, theranosis

## Abstract

Cancer is the second leading cause of death in the world, which is why it is so important to make an early and very precise diagnosis to obtain a good prognosis. Thanks to the combination of several imaging modalities in the form of the multimodal molecular imaging (MI) strategy, a great advance has been made in early diagnosis, in more targeted and personalized therapy, and in the prediction of the results that will be obtained once the anticancer treatment is applied. In this context, magnetic nanoparticles have been positioned as strong candidates for diagnostic agents as they provide very good imaging performance. Furthermore, thanks to their high versatility, when combined with other molecular agents (for example, fluorescent molecules or radioisotopes), they highlight the advantages of several imaging techniques at the same time. These hybrid nanosystems can be also used as multifunctional and/or theranostic systems as they can provide images of the tumor area while they administer drugs and act as therapeutic agents. Therefore, in this review, we selected and identified more than 160 recent articles and reviews and offer a broad overview of the most important concepts that support the synthesis and application of multifunctional magnetic nanoparticles as molecular agents in advanced cancer detection based on the multimodal molecular imaging approach.

## 1. Introduction

Cancer is currently one of the leading causes of death worldwide. According to the statistics reported by World Health Organization (WHO), it is the second leading cause of death in the world after cardiovascular disease, accounting for nearly 10 million deaths in 2020 [1]. Neoplastic diseases are very complex pathologies characterized by a very heterogeneous context with common hallmarks [2] and an uncontrolled cell division with abnormal cell morphology and/or function [3,4]. Current treatments are based on a combination of chemotherapy, radiation, and surgery, but they are mainly focused on treating local and non-metastatic cancers and often lead to severe side effects. Due to the limitations of conventional therapies, the development of new strategies able to maximize the therapy efficiency and minimize the non-desirable toxic side effects is under investigation [5,6].

Furthermore, even with the improvements in diagnosis, only a small part of the population with cancer is diagnosed at the early stages of the disease due to the low selectivity and sensitivity of traditional diagnostic techniques. In fact, traditional bioimaging techniques mostly detect anatomical alterations that differentiate pathological from healthy tissue, rather than measuring the biological processes responsible for disease. Conversely, an early cancer diagnosis will facilitate the most efficient and effective management of cancer treatment. Within this scope, molecular imaging (MI)—or the combination of in vivo biomedical imaging and molecular biology—has appeared as a strong biomedical research system enabling the visual representation, characterization, and quantification of biological procedures at the subcellular and/or molecular levels within living organisms in a noninvasive way [7,8,9]. Therefore, molecular imaging modalities can identify the cancer mass based on its physical properties, which is highly beneficial for individualized diagnosis and for the prediction and follow-up of cancer therapy.

**Figure 1:**
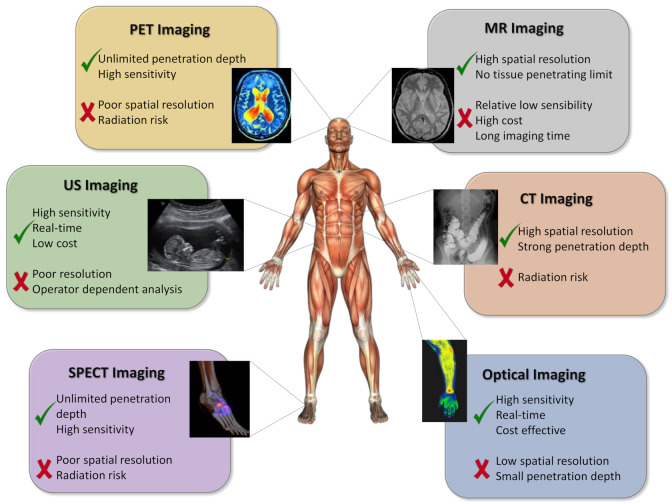
Schematic representation of different molecular imaging modalities. Adapted with permission from [15], Copyright 2021 Elsevier Ltd.

There are several imaging modalities that, as a result of the intrinsic physical characteristics, provide different information for pathological tissues, such as structural, functional, and molecular information. Currently, the most useful imaging techniques in cancer preclinical research and the clinical field are: magnetic resonance imaging (MRI, based on the principle of nuclear magnetic resonance), computed tomography (CT, which provides images reflecting human anatomy and is based on the different attenuation of X-rays by different biological tissues), ultrasound imaging (US, which uses the propagation of high-frequency sound waves through different organs to provide morphological and anatomical information), positron emission tomography and single-photon emission computed tomography (PET and SPECT, both based on particular radionuclides included in relevant biologic molecules and delivered to the body, so that their uptake and metabolization can be measured), and optical imaging (OI, based on the detection of optical molecules as imaging biological probes) [10,11,12,13,14]. Individually, all the imaging techniques described above present certain limitations, such as low sensitivity and spatial resolution or limited tissue penetration, which are related to the fundamental characteristics that enable the retrieval of key information when diagnosing and treating a disease. The advantages and disadvantages of these techniques due to their different physical natures are summarized in Figure 1.

To compensate for the weaknesses of each single traditional molecular imaging modality, the merging of two or more different imaging techniques—the multimodal molecular imaging approach—has emerged as a powerful strategy. Furthermore, consistent and accurate information can be achieved with this approach, providing significant improvements in presymptomatic detection, targeted therapy, and personalized medicine [13]. Multimodal molecular imaging has had an impact in preclinical and clinical research. The mixing of different imaging modalities in dual or even triple modes makes it possible to overcome the inherent shortcomings of each one, thus greatly enhancing the diagnosis of and therapy for different diseases. For example, the merging of anatomical and molecular modalities can make it possible to specifically measure the staging of the disease (whether it is metastatic or not) and predict therapy or surgery reactions before their execution [16]. Therefore, more individualized medicine can be achieved [17]. From a stricter diagnostic point of view, multimodal molecular imaging can increase early diagnosis, as more sensitivity and resolution can be achieved. For example, biomarkers or injuries in a certain area can be identified in a subclinical stage, thus allowing the early prognosis of primary tumors or possible metastases [18]. Despite its highly experimental stage, from a translational point of view, the multimodal molecular imaging approach has already found clinic applications. For example, Unak et al. combined CT and PET modalities through the conjugation of AuNPs with the ^18^F-FDG molecule and an antibody that specifically targets breast cancer cells, achieving with this unique probe an increase in tumor tissue contrast (with CT) and information about the increase in glucose metabolism (with PET), which is typical in cancer cells. This multiparametric achievement could not have been obtained with one single technique [19]. In light of the mentioned benefits, the multimodal approach is poised to radically transform the diagnosis of and therapy for neoplastic and various other diseases (Table 1).

The implementation of a single imaging modality, or its combination with others, requires research on specific, sensitive, and targeted contrast agents. In this context, multifunctional nanoparticles (NPs) have appeared as a new type of MI agent able to overcome the main problems in the diagnosis of cancer diseases [20,21,22,23,24]. The use of nanomaterials is highly beneficial because of their tunable size, shape, and surface characteristics. As a result, they demonstrate high stability, large carrier capacity, the ability to incorporate both hydrophilic and hydrophobic molecules, and compatibility with different administration routes, this last advantage being a crucial parameter that makes them highly desirable agents in many aspects of medicine.

Thanks to their biocompatibility, biodegradability, and physiological stability, iron oxide nanoparticles (IONPs, magnetite Fe_3_O_4_ or maghemite γ-Fe_2_O_3_) have become one of the first choices for cancer diagnosis (mediated by MRI) and therapy (mediated by magnetic hyperthermia). IONPs have the great advantage that they can be easily functionalized with different molecules, allowing specific MI modalities as a function of their constituting material and permitting the precise delivery of their cargo to the pathologic tissue upon systemic administration [25,26]. Nevertheless, as previously stated, an individual molecular imaging technique cannot offer all the requirements for an optimal in vivo application. For example, MRI can provide anatomical and physiological images with high spatial resolution, but it does not show enough sensitivity. While optical or radionuclide molecular imaging techniques provide physiological and molecular images with high sensitivity, they have limited tissue penetration depth and relatively poor spatial resolution, respectively. As a result, multimodal imaging can compensate the deficiencies of certain modalities with the advantages of others, thus expediting the progress of nanomaterials to clinical trials and providing key data at the preclinical phase [27]. Therefore, current approaches to multimodal molecular imaging consist of designing multifunctional contrast agents made up of different entities that allow improvements to the contrast of two or more imaging techniques simultaneously. Actually, PET–CT is the only multimodal molecular imaging strategy that has demonstrated clinical translatability. Toward this end, Philips, for example, one of the world leaders in clinical medical imaging, has developed a mixed PET–CT scanner that is used in clinics for cancer diagnosis [28]. The other dual modalities, such as MRI–optical, MRI–PET/SPECT, MRI–CT, MRI–MPI, MRI–MMUI, and MRI–MPA, are still in preclinical phases and further investigations should be undertaken, but they have shown promising results that are discussed in the next sections of this review. To understand how multifunctional magnetic nanoparticles can be employed in therapy for and diagnosis of cancer, some fundamental notions of cancer physiology (e.g., the EPR effect or active targeting) should be kept in mind. Toward this end, some elegant and instructive publications can be found in the literature [2,29,30,31,32,33].

Thus, in this review, we offer a wide and up-to-date overview regarding the use of iron-based magnetic nanoparticles as contrast agents in MRI alone or in combination with different molecular agents in order to allow multimodal molecular imaging of cancer diseases [23,34,35], thus improving the characteristics and overcoming the disadvantages of individual traditional imaging techniques. In more detail, this review is intended to inform the reader about the importance of combining different imaging techniques, as well as about the development of multimodal contrast agents that have achieved a great advance in the detection and treatment of cancer thanks to the multimodal molecular imaging approach.

## 2. Magnetic Nanoparticles as Molecular Imaging Agents in Magnetic Resonance Imaging

Magnetic resonance imaging (MRI) is an extremely robust and flexible imaging technique that makes it possible to obtain three-dimensional images with great resolution through the application of a magnetic field and radio frequency (RF) pulses. MRI registers the proton relaxation times, which are contained in water, proteins, and lipids, and generates good spatial resolutions with excellent endogenous contrast, without the need for ionizing radiation or radioisotopes [36]. Considering that MRI retrieves exceptionally clear and detailed anatomical and functional images of soft tissues, it has become an essential and noninvasive technology for clinical diagnosis and treatment of cancer. In addition, it can establish whether a tumor has proliferated and is thus also extremely useful for the follow-up of the selected treatment. MRI has been demonstrated to be especially effective for imaging certain liver cancers, soft tissue sarcomas, primary bone tumors, brain metastases, and tumors affecting the spinal cord and the pelvic organs [37,38,39]. Different contrast agents can be orally or intravenously administered to improve the images and visibility of internal organs and tissues structures in MRI [40].

As a general consideration, MRI contrast agents can be divided into two groups according to their influence on the two proton relaxation processes: T1-weighted or positive contrast agents and T2-weighted or negative contrast agents. T1 probes reduce the longitudinal relaxation time, which generates a brighter contrast (hyperintense signals), whereas T2 probes decreases the transverse relaxation time, producing a darker contrast (hypointense signals) [36]. The r_2_/r_1_ ratio (r_1_ and r_2_ are the relaxation rates and correspond to the inverse of T1 and T2, respectively) indicates the contrast effectiveness; it has been determined that an r_2_/r_1_ ratio lower than 2 corresponds to T1 contrast agents and one greater than 10 corresponds to T2 contrast agents, while intermediate values are considered for potential dual T1–T2 contrast agents [41].

Paramagnetic complexes of transition as well as lanthanide metals (Gd^3+^ and Mn^2+^) are commercially available and can act as T1 contrast agents [36]. The disadvantages of these complexes are that they have poor sensitivity and cannot be used in molecular imaging applications. Therefore, iron oxide nanoparticles (IONPs) have been widely investigated as alternative contrast agents to overcome these limitations [42,43]. For example, Feridex^®^ (commercial IONPs coated with dextran) has been clinically approved for the detection of various diseases as a T2 contrast agent [18]. IONPs present many advantages, such as elevated sensitivity and biocompatibility and extended blood circulation. These characteristics depend on their structural properties and overall design, as well as their synthetic qualities [40,44]. Therefore, magnetic nanoparticles can act as efficient T1 or T2 magnetic resonance contrast agents by accurately setting up and optimizing their r_1_ and r_2_ behavior and, in consequence, their r_2_/r_1_ ratios [18].

### 2.1. Characteristics of Magnetic Nanoparticles Impacting T1 and T2 MRI Signals

In general, the effectiveness of magnetic nanoparticles as contrast agents—mainly related to their relaxometric properties—are determined both by structural parameters (size, shape, composition, and crystallinity) and by surface parameters (characteristics of the coating and state of aggregation) [36,41]. Several exhaustive reviews have been published focusing on these aspects [18,45]. Nonetheless, the most relevant characteristics to be considered for the application of IONPs as MRI contrast agents are summarized below:

**Size and shape.** An increase in particle size leads to an improvement in T2-weighted contrast [40,45], while a reduction in particle size (below 3 nm) produces an increase in T1-weighted contrast [40,42,43,46]. Regarding the shape, it has been found that non-spherical nanoparticles lead to a larger effective radius with respect to the equivalent spherical particles, which results in a greater relaxation of T2 [45,47];

**Crystallinity and composition.** In general, the better the nanoparticle crystallinity, the greater the T2 relaxation process [45]. Moreover, IONPs’ magnetic properties can be controlled by varying their composition—for example, by doping with other elements—which also impacts the T1 and T2 relaxation values [40,48];

**Surface modification and aggregation state**. The coating nature strongly impacts the nanoparticles’ behavior as MRI contrast agents. In fact, the thickness of the nanoparticle is inversely proportional to the relaxation rate [40,41]. On the other hand, the aggregation of controlled IONPs is also an approach that has been used to enhance r_2_ relaxation [40].

### 2.2. T1–T2 Dual-Mode MRI Contrast Agents

**Figure 2:**
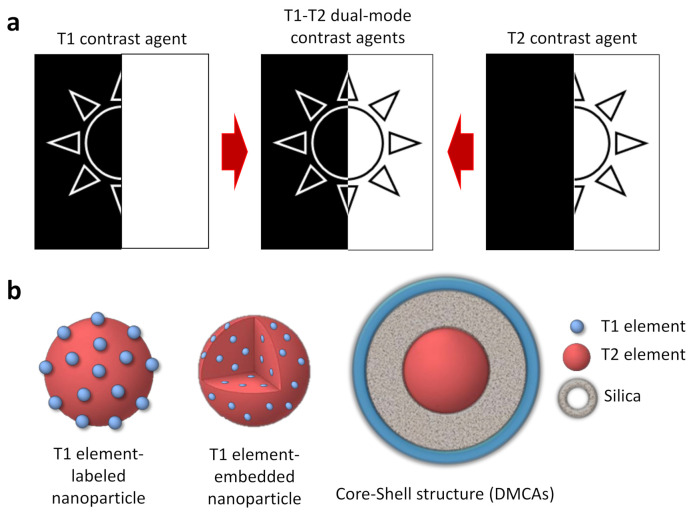
(**a**) Schematic representation of T1, T2, and T1–T2 dual contrast effects; (**b**) schematic illustration of three possible structures for a T1–T2 dual-mode contrast agent. Adapted with permission from [15], Copyright 2021 Elsevier Ltd.

To obtain more precise MRI images, dual contrast agents can be prepared by combining T1 and T2 contrast effects. In this way, self-confirmation is achieved with a better distinction between normal and pathological regions (Figure 2a) [49,50]. The simplest method to construct T1–T2 dual-mode contrast agents is the coupling of T1 and T2 elements in the same entity. For example, different Gd compounds (T1 contrast agents) have been attached to or embedded in iron oxide magnetic nanoparticles (T2 contrast agents) (Figure 2b) [18,51,52,53]. Another study describes a similar T1–T2 dual-mode effect in the case of europium-doped iron oxide nanoparticles [54].

However, this conjugation strategy is not the most appropriate for obtaining dual-mode T1 and T2 contrast agents, as different problems can arise due to the strong magnetic coupling between the T1 and T2 effects. To overcome this difficulty, magnetically decoupled T1–T2 dual-mode contrast agents (DMCAs) have been developed [18,49]. Generally, these systems are based on a core-shell-type structure with the T2 component located in the center and the T1 component exposed on the surface. Furthermore, to prevent magnetic coupling, it is necessary to introduce a non-magnetic separating layer between both components (Figure 2b).

### 2.3. Applications of MRI Nanomaterials in Cancer Diagnosis

Since the 1990s, numerous iron-based MRI contrast agents have been developed. These materials have been extensively used for diagnostic purposes, in particular for visualizing tumors and metastases in liver, spleen, and lymph nodes [55]. So far, only five IONPs have been clinically approved for MRI. These products are Feridex^®^, Resovist^®^, Combidex^®^, Gastromark^®^, and Feraheme^®^ [56]. However, most of the mentioned products have been removed from the market due to either safety concerns or the absence of benefits [49]. Various elegant reviews of the recent advances in the development of IONP contrast agents and their applications in biomedicine can be found in the literature [25,36,37,44].

#### 2.3.1. MRI Nanomaterials as T2 Contrast Agents in Cancer Imaging

**Figure 3:**
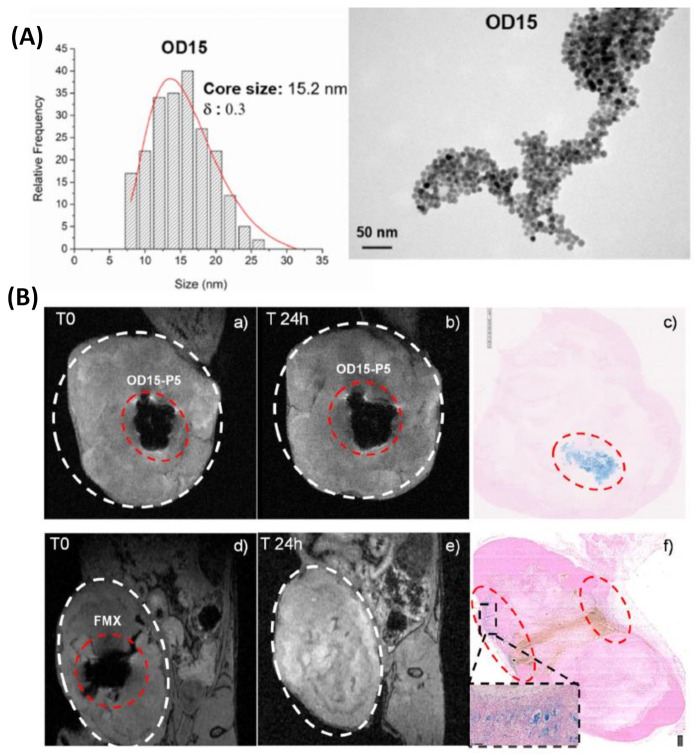
(**A**) Size distribution with log-normal fit (left) and TEM micrograph (right) of OD15 sample. (**B**) Coronal view for T2-weighted gradient-echo 3D MRI of the tumor region in a mouse after the injection of IONPs ((**a**), OD15-P5 and (**d**), Ferumoxytol) and 24 h post-injection ((**b**), OD15-P5 and (**e**), Ferumoxytol), showing Prussian blue staining of tumor section 24 h post-injection ((**c**), OD15-P5 and (**f**), Ferumoxytol). Figure adapted with permission from [61], Copyright 2019 Elsevier B.V.

Due to the great usefulness of the MRI technique in clinical diagnosis and the tremendous potential of IONPs, the proper functionalization of the latter (e.g., with epithelial growth factor receptor, antibodies, short peptides sequences, or aptamers, among others) as an advanced contrast agent in MRI-mediated molecular imaging has enabled the targeted diagnosis of various types of cancer, including breast, stomach, colon, kidney, liver, and brain cancer [44]. Consequently, a series of promising applications have emerged in recent years [57,58,59]. For example, Chee et al. published an attractive study describing the design of a short peptide and ligand library to functionalize IONPs. Thanks to this library, they were able to choose the ligand that provided IONPs with the most advantageous features for in vivo applications, finally obtaining a considerable enhancement in contrast between the liver tumor and the healthy liver tissue in comparison with a commercial MRI contrast agent [60]. In another interesting example, Lazaro-Carrillo and co-workers developed fine-tuned iron oxide nanoparticles (IONPs) coated with polyethyleneglycol and useful in MRI cancer imaging. In vitro characterization of the PEG–IONPs revealed high biocompatibility and r_2_ relaxivity values greater than the commercial alternative Ferumoxytol. The in vivo characterization with breast cancer mouse models showed the capability of these PEG-coated IONPs to be totally preserved at the tumor site for up to 24 h, thus indicating their strong potential as MRI contrast agents for real-time long-lasting monitoring of tumor progression [61] (Figure 3).

**Figure 4:**
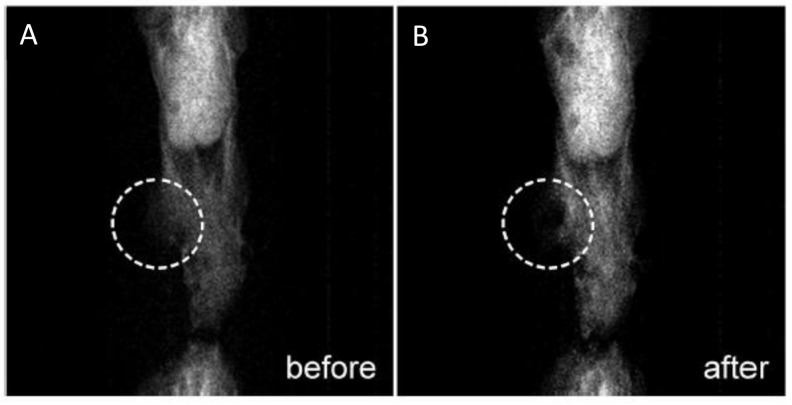
In vivo MR images of a mouse (**A**) before and (**B**) after injection of BPs@Au@Fe_3_O_4_ nanocomposite. Figure adapted with permission from [62], Copyright 2017 WILEY-VCH Verlag GmbH & Co. KGaA, Weinheim.

Yang et al. developed a new nanocomposite by assembly of iron oxide (Fe_3_O_4_) and Au nanoparticles on black phosphorus sheets (BPs@Au@Fe_3_O_4_). This nanostructure can anticipatorily suppress tumor growth through visualized synergistic treatment with the help of magnetic resonance imaging (MRI) due to the combination of the photodynamic effect of BPs, the photothermal effect of Au nanoparticles, as well as the tumor-targeting and MRI-guiding abilities of iron oxide nanoparticles (Figure 4) [62].

#### 2.3.2. MRI Nanomaterials as T1 Contrast Agents in Cancer Imaging

**Figure 5:**
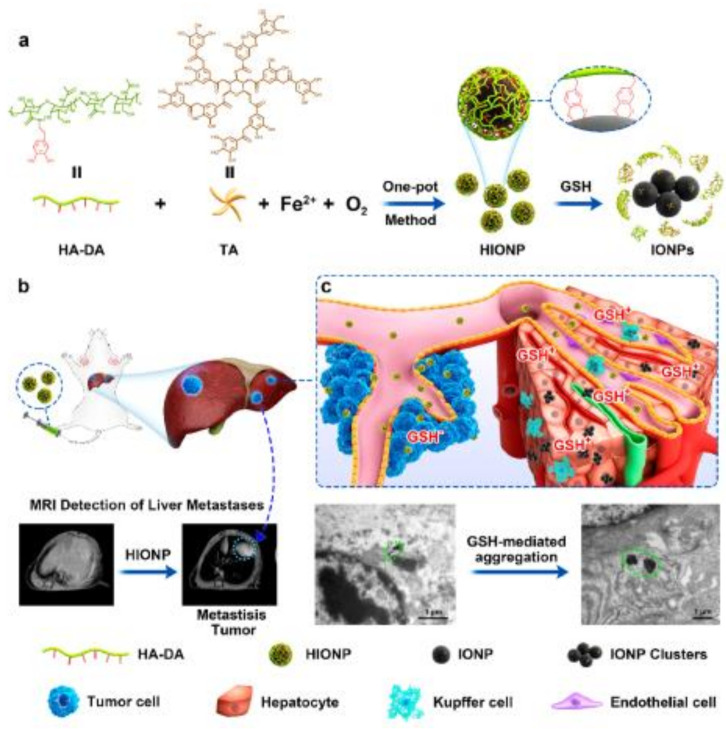
(**a**) Schematic illustration of the production of HIONPs using a one-pot method and the GSH-responsive transformation; (**b**) schematic illustration of GSH-responsive magnetic HIONPs for exceedingly sensitive diagnosis of liver metastases; and (**c**) GSH-mediated conversion of HIONPs in metastasis tumor (non-aggregation state) and liver (aggregation state). Figure adapted with permission from [64], Copyright 2021 American Chemical Society.

In a recent study, a GSH-activatable contrast agent consisting of ICNs-RGD, with a modulatable MRI signal from T2 to T1 and a tumor targeting function, was developed through encapsulation of IONPs-CA in zwitterionic poly-(CBMA) nanogels and the further introduction of a c(RGD) ligand. Under the stimulus of GSH, ICNs-RGD can be effectively dissociated, and the clustered IONPs convert into dispersed ones. Based on this, ICNs-RGD can switch from a T2 contrast agent to a T1 one, thus allowing the activation of T1 contrasting enhancement [63]. A similar approach based on the GSH response was carried out by Xu et al. for the early and accurate diagnosis of liver metastasis [64]. They prepared glutathione (GSH)-responsive hyaluronic acid-coated iron oxide nanoparticles (HIONPs) for exceedingly sensitive diagnosis of LMs through a simple one-pot method. HIONPs significantly improve the signal of MRI in tumor metastases as a T1 contrast agent (CA), whereas they substantially reduce the signal for the liver as a T2 CA, as they aggregate into clusters in response to the high GSH in the liver. Consequently, MRI contrasted by HIONPs clearly differentiates metastatic tumors (bright) from nearby liver tissues (dark) (Figure 5).

#### 2.3.3. MRI Nanomaterials as T1–T2 Contrast Agents in Cancer Imaging

Motivated by the natural binding capacity of polydopamine nanospheres (PDAs) with iron ions, an easy and flexible synthesis approach was developed by Chen and coworkers to produce biodegradable coordination polymer (CP)-encapsulated PDA nanocomplex (PDAs@CPx, x = 3, 6, 9). They found that the PDAs@CP3 can serve as a T1/T2 dual mode contrast agent (DMCA) for magnetic resonance imaging (MRI) in a hepatocellular carcinoma murine model, which possessed high longitudinal (r_1_ = 7.524 mM^−1^ s^−1^) and transverse (r_2_ = 45.92 mM^−1^ s^−1^) relaxivities [65].

Shu et al. obtained SAPEG-MPDA@SPIO/DOX/Fe^3+^ NPs (mesoporous polydopamine NPs combined with superparamagnetic iron oxide NPs, modified with a targeted molecule of sialic acid, chelated with Fe^3+^, and loaded with doxorubicin drug) as a new theranostic agent for T1/T2 dual-mode MRI-guided chemo-photothermal treatment of liver cancer. They found that SAPEG-MPDA@SPIO chelated with Fe^3+^ had excellent T1 positive- and T2 negative-contrast improvement effects [66].

**Figure 6:**
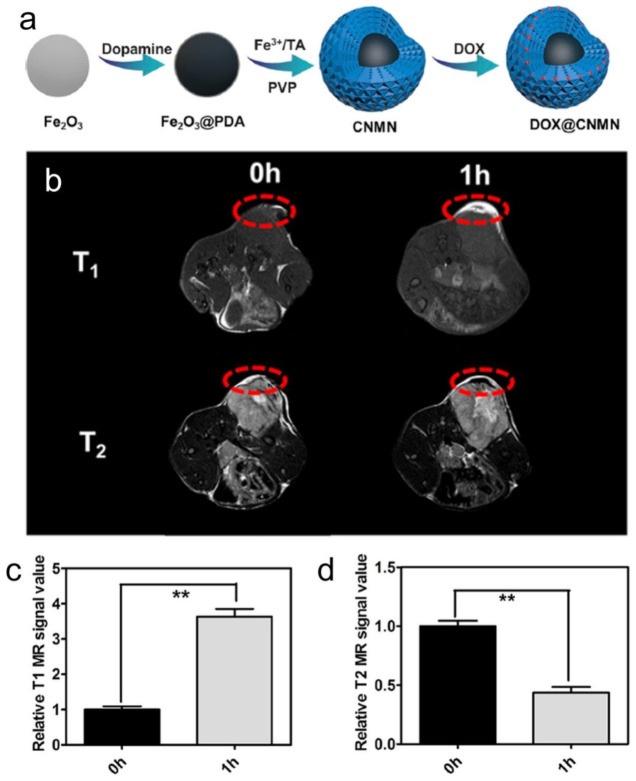
(**a**) Schematic illustration of the preparation of DOX@CNMN; (**b**) T1/T2 MR images of mice; and (**c**,**d**) the corresponding data analysis after intravenous injection of CNMN nanoparticles at different time intervals. The time of 0 h means pre-injection (*n* = 3). ** *p* < 0.01. Figure adapted from [70]. Copyright 2020 American Chemical Society.

Another interesting dual-mode T1–T2 contrast agent is composed of a 15 nm MnFe_2_O_4_ nanoparticle core as the T2 contrast material covered with a 1.5 nm Gd_2_O(CO_3_)_2_ external layer as the T1 contrast material. The magnetic decoupling separation between the two materials is controlled by using a precise 16 nm SiO_2_ layer [67,68,69]. Recently, Li et al. obtained an avocado-like Fe^3+^/Fe_2_O_3_-composed T1–T2 dual-mode contrast agent based on the Fe^3+^-tannic acid (TA) coordination network (CNMN). This material possesses appropriate longitudinal and transverse relaxation values. The Fe–TA complex was studied as the loose flesh and Fe_2_O_3_ as the hard core of this avocado-like nanoparticle. Specifically, Fe–TA was adhered to polydopamine-modified Fe_2_O_3_ (Fe_2_O_3_@PDA), which was further coated with PVP to form the coordination network magnetic nanoparticles. The PDA layer could function as a separator to avoid the undesired interference between T1 and T2 imaging (Figure 6) [70]. In another report, ultrasmall Fe@Fe_3_O_4_ nanoparticles were designed and synthesized as T1–T2 dual-mode MRI contrast agents for accurate tumor imaging. The 8 nm 3-(3,4-dihydroxyphenyl)propionic acid (DHCA)-modified nanoparticles exhibited optimal T1–T2 dual-mode MRI performance. Next, to develop a tumor-targeted contrast agent, the DHCA-Fe@Fe_3_O_4_ nanoparticles were conjugated with the F56 peptide—which targets the vascular endothelial growth factor receptor—and the resulting F56-DHCA-Fe@Fe_3_O_4_ nanoparticles were found to exhibit good T1–T2 dual-mode imaging and tumor-targeting performance both in vitro and in vivo [71].

Recently, Wang et al. reported a UHF-tailored T1–T2 dual-mode iron oxide nanoparticle-based contrast agent (UDIOC) with extremely small core size and ultracompact hydrophilic surface modification, exhibiting dually enhanced T1–T2 contrast effects under the 7 T magnetic field. The UDIOC enables clear visualization of microvasculature at sizes as low as ≈140 µm in diameter under UHF MRI, extending the detection limit of the 7 T MR angiography. Moreover, by virtue of the high-resolution UHF MRI and a simple double-checking process, UDIOC-based dual-mode dynamic contrast-enhanced MRI has been successfully applied to detect tumor vascular permeability with extremely high sensitivity and accuracy [72].

## 3. Hybrid Magnetic Nanoparticles as MRI–Optical Dual-Mode Imaging Agents for Cancer Diagnosis

As stated above, various imaging modalities can be coupled to improve cancer diagnosis. One important example is represented by the synergy between MRI and the optical imaging modality, where compounds with optical properties bind (physically or chemically) to nanoparticles with magnetic properties to promote both molecular imaging modalities.

This combination of MRI with optically active compounds compensates for the disadvantages of both techniques by generating a single multifunctional nanoplatform, which is suitable for in vivo biomedical imaging studies [73]. Thus, by combining the advantages of the two imaging modalities, it is possible to obtain images with high sensitivity and high temporal and spatial resolution [18].

In addition to widely used chemical fluorochromes, the association of quantum dots (QDs) with magnetic nanoparticles has been shown to enhance their fluorescence due to their high photostability, high extinction coefficients, and excellent absorption properties [74,75,76]. In general, three methods have been described for the synthesis of these hybrid nanoplatforms that exhibit both magnetic and fluorescent properties: covalent bonding, silica matrix encapsulation, and dispersion in nanoassemblies [76]. Some representative studies are listed in Table 2:

Some representative examples of each synthetic strategy are described below.

**Figure 7:**
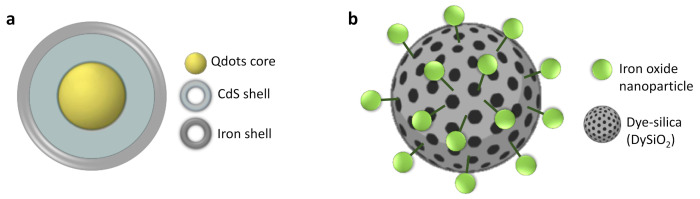
General strategies for MRI–OI dual agent synthesis based on the association approach. (**a**) Core–shell nanoparticles; (**b**) core–satellite. Adapted with permission from [15], Copyright 2021 Elsevier Ltd.

**Association by covalent bonding (nanoparticles).** Core–satellite systems can be obtained by covalent bonding. Pahari et al. [75] used a reverse strategy to encapsulate a fluorescent QD core inside a hollow iron oxide sphere acting as an NMR contrast agent (Figure 7a). This hollow morphology allows the combination of both imaging techniques in a single structure, while avoiding direct contact between each species and preventing the quenching of optical fluorescence.

Using a similar design, hybrid ”core–satellite” nanosystems have been synthesized by functionalizing fluorescent silica nanoparticles with iron oxide nanoparticles (Figure 7b) [78].

**Figure 8:**
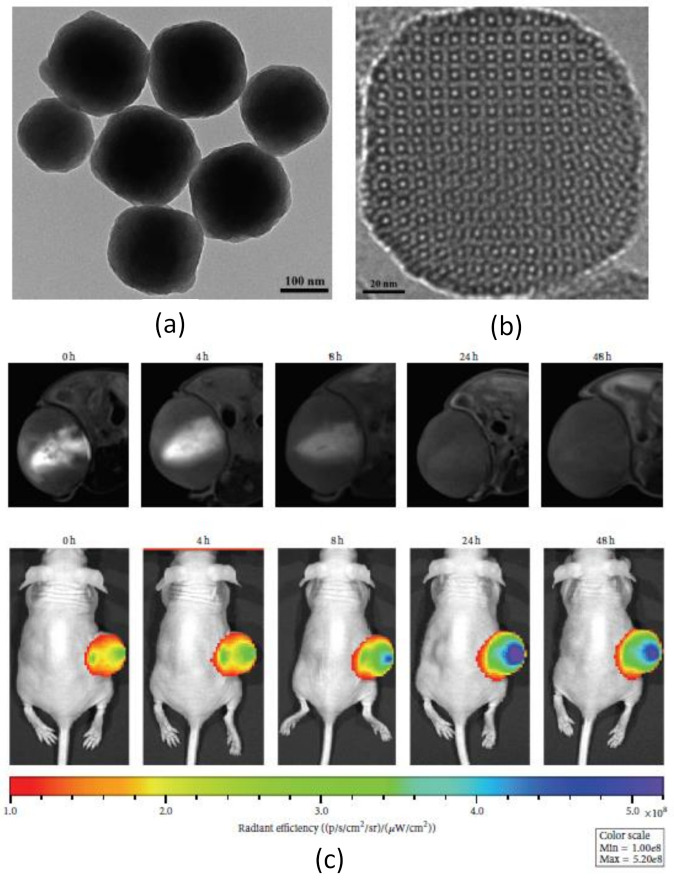
(**a**,**b**) TEM images of the aminated mesoporous silica nanoparticles synthesized by CTAB-directed sol-gel processes using TEOS and APTES as co-precursors; (**c**) in vivo dual-modality imaging (MR and NIR fluorescence) of U87-MG tumor-bearing mouse model after intratumoral injection of Gd/NIR-MSNs at 0, 4, 8, 24, and 48 h. Figure adapted from [79], Copyright 2016 Hindawi Limited.

**Encapsulation in silica matrix (nanostructures).** The incorporation of magnetic nanoparticles and fluorescent moieties into mesoporous silica particles has been carried out to protect the particles and fluorescent molecules from the surrounding medium [76]. Zhu et al. [79] synthesized mesoporous silica nanoparticles (MSNs) with functionalized amine groups on their surface to create a multifunctional nanoplatform with a near-infrared dye and a contrast agent, Gd-DTPA (Figure 8). After intratumor injection with the dual-modality imaging probe, MRI contrast makes it possible to see the tumor region with greater definition, while NIR fluorescence imaging significantly increases the tumor detection capability, thus enabling a more sensitive diagnosis that provides great potential in biomedical imaging.

**Figure 9:**
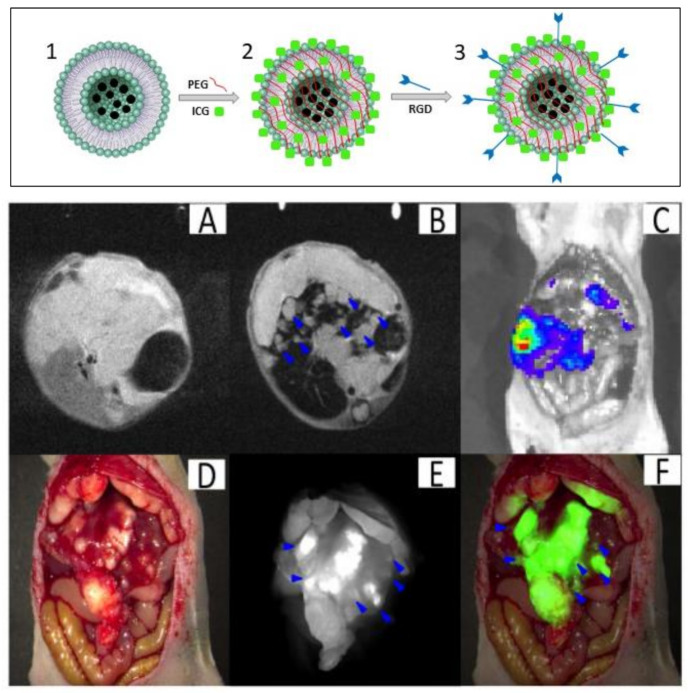
Synthesis of SPIO@liposome-ICG-RGD (upper panel). (1) SPIO nanoparticles encapsulated in liposomes. (2) Magnetoliposome functionalized with ICG molecules. (3) Conjugation of RGDs peptide; orthotopic liver tumors with intrahepatic metastasis (lower panel). (**A**) The MRI image before SPIO@liposome-ICG-RGD injection. (**B**) The MRI signal was decreased in normal liver tissue (CNR: 14.6 ± 9.9) after targeting probe injection and the disseminated tumor nodes (0.9 ± 0.5 mm) (blue arrows) can be clearly defined. (**C**) Liver tumors confirmed by bioluminescence imaging. (**D**) Surgical guidance by intraoperative FMI-NIR. (**E**) The implanted liver tumor tissue (0.7 ± 0.3 mm) (blue arrows) exhibits obvious contrast (TBR: 2.3 ± 0.5) in colour and texture with normal liver tissues. (**F**) Merged colour and fluorescence image. Figure adapted from [94], Copyright 2017 Oncotarget.

**Dispersion in nanoassemblies (supramolecular assembly).** Several studies describing different types of molecular matrices composed of polymers [80,81,82,83,84,86,87,88], lipids [76], polymer dots (PDots) [89,90], and organic molecules [91,92,93] can be encountered in the literature. For example, Chen et al. [94] encapsulated superparamagnetic iron oxide nanoparticles (SPIONs) into liposomes containing PEG, a tumor-targeted peptide (RGD sequence), and ICG (indocyanine green, the only near-infrared dye approved by the Food and Drug Administration (FDA) in the United States for clinical use) (Figure 9) [95]. The hybrid nanosystem was able to detect small tumors thanks to the MRI technique, which is effective for preoperative diagnosis. Furthermore, active targeting and a long clearance rate allowed fluorescence imaging to detect small tumor metastasis lesions [95].

**Figure 10:**
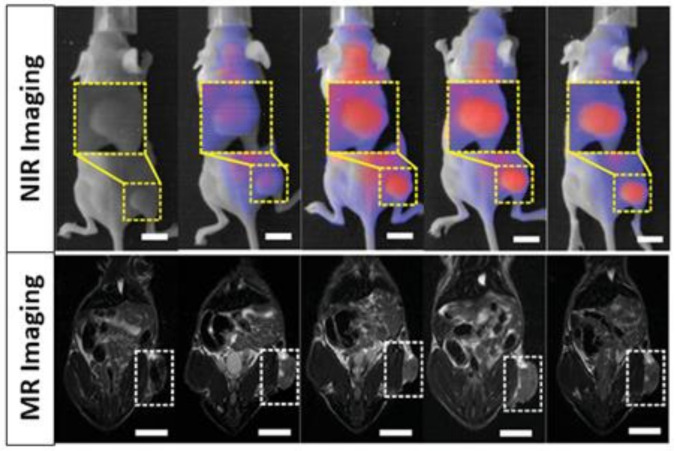
NIR and MRI mice images after administration of Janus nanoparticles. Adapted with permission from [96], Copyright 2021 Wiley-VCH GmbH.

In another approach, Lin et al. [96] designed glutathione-sensitized nanoplatforms that are able to modify their size and adjust their radiosensitisation to perform imaging-guided radio- and chemotherapy. These systems were synthesized by attaching gold and manganese oxide Janus nanoparticles that encapsulated fluorescent molecules capable of emitting in the near-infrared window. Once these particles come in contact with glutathione, they dissociate into smaller gold nanoparticles and manganese ions that are able to be internalized into the tumor and develop a chemotherapeutic response, thereby increasing the efficacy of the therapy. Thanks to the presence of these fluorescent molecules and manganese, a very precise detection of the tumor is possible at the same time as treatment, creating a promising *theranostic* (therapy plus diagnostic) agent (Figure 10).

**Figure 11:**
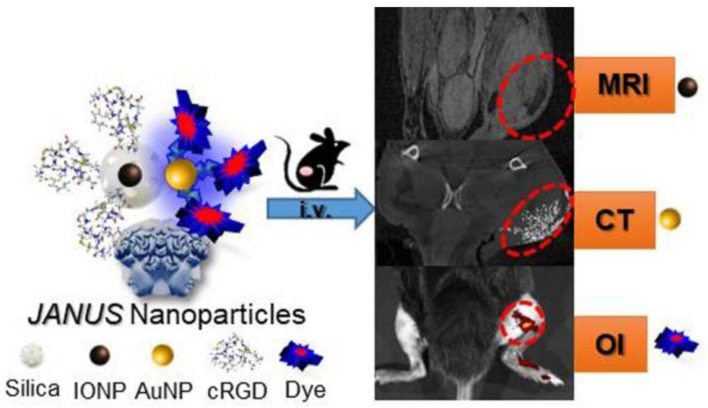
Janus nanoparticle for multimodal molecular imaging of solid cancer by MRI, CT, and optical imaging. Adapted with permission from [97], Copyright 2018 American Chemical Society.

Owing to its intrinsic synthetic flexibility, it is also possible to prepare a nanosystem that combines more than two different types of imaging modalities. In their study, Sanchez et al. [97] synthesized a double-sided Janus nanoparticle that encapsulates iron oxide nanoparticles into a mesoporous silica nanoparticle on one side and a gold nanoparticle on the other side (Figure 11). This combination results in a multifunctional nanosystem able to express three different imaging modalities: MRI (iron oxide core), computed tomography (CT) (gold nanoparticle), and optical imaging (fluorochrome). As a result, a nanoplatform for precise detection of fibrosarcoma in murine models was successfully obtained.

## 4. Hybrid Magnetic Nanoparticles for MRI Radiation-Based Dual-Mode Imaging for Cancer Diagnosis

Another important multimodal imaging approach can be enhanced through the merging of radioisotope/X-ray-based imaging modalities (PET/SPECT/CT) with MRI. These combinations can offer high-resolution tomographic images along with robust sensitivity, enabling precise determination of quantitative biological information such as in vivo biodistribution and pharmacokinetics, particularly in deep tissues [18]. Some representative examples for each multimodal imaging combination are discussed below.

### 4.1. MRI–PET/SPECT Dual Imaging

Nuclear imaging techniques (PET or SPECT) and MRI are each well-established imaging modalities used in preclinical and in clinical stages. PET/SPECT offers exceptional sensitivity but poor spatial resolution, while MRI can provide excellent spatial resolution. The MRI–PET/SPECT combination also requires less exposure to doses of radiation, thus addressing another important weakness of the radionuclide modalities. Moreover, this dual-mode approach can be used without a limited tissue penetration depth, which is a challenge that appears in other modalities such as optical imaging [10,98].

The development of imaging agents for the radionuclide–MRI combination is usually achieved by coupling magnetic nanoparticles with radioactive isotopes on the surface or within their core. These radioactive isotopes are different depending on each nuclear technique, and typically ^18^F, ^64^Cu, ^68^Ga, and ^124^I are used for PET and ^99m^Tc, ^111^In, and ^131^I are used for SPECT [13,99,100].

#### 4.1.1. MRI–PET Dual Imaging in Cancer Diagnosis

Whole-body MRI–PET scanners for clinical practice are currently commercially available [98]. MRI provides excellent anatomical images with enhanced spatial resolution, which is one of the disadvantages of PET; on the other hand, PET provides high sensitivity, bringing both structural and molecular or functional information [101].

**Figure 12:**
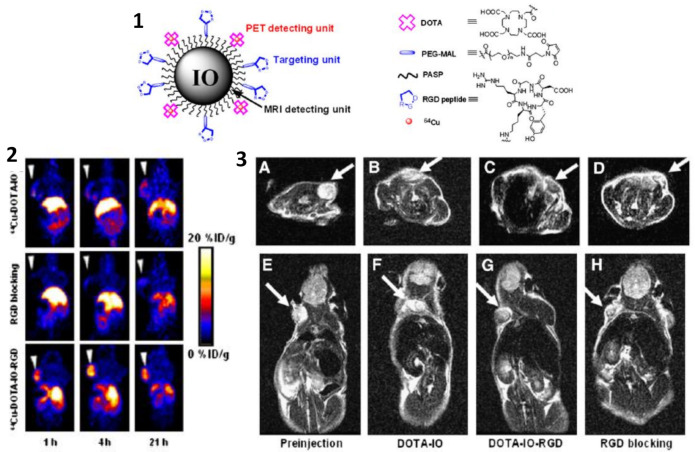
(**1**) Illustration of PET/MRI probe based on iron oxide nanoparticle. (**2**) Decay-corrected whole-body coronal PET images of nude mouse bearing human U87MG tumor at 1, 4, and 21 h after injection of different PET/MRI probes based on iron oxide nanoparticles. (**3**) T2-weighted MR images of nude mice bearing U87MG tumor before injection of iron oxide nanoparticles (**A**,**E**) and at 4 h after tail-vein injection of DOTA-IO (**B**,**F**), DOTA-IO-RGD (**C**,**G**), and DOTA-IO-RGD with blocking dose of c(RGDyK) (**D**,**H**). Figure adapted from [103], Copyright 2008 Society of Nuclear Medicine, Inc.

Preclinical approaches in cancer management have been assessed in this context. For example, Goldenberg et al. showed that the application of dual PET–MRI generated a better evaluation of a therapeutic reaction in pancreatic cancer animal models [102]. In another example, Lee et al. targeted a tumor with integrin ανβ3 expression with iron oxide nanoparticles (MRI) combined with a ^64^Cu radionuclide (PET) through a DOTA chelator and using arginine–glycine–aspartic (RGD) peptide as targeting agent. They demonstrated with both modalities that these conjugates bound exclusively to integrin ανβ3 in vitro, thus showing the great usefulness of this dual-imaging method for tumor detection with high accuracy (Figure 12) [103].

Nevertheless, with regard to clinical oncology applications, it is worth mentioning that nowadays the PET–CT imaging technique is still the gold standard; hence, the benefits of the MRI–PET combination should be demonstrated. Within this scope, one of the most important advantages of MRI–PET is the high contrast of soft tissue for MRI combined with PET data, which can be very useful in characterization and biopsy planning for primary tumors, such as prostate, lung, or breast cancer [104,105,106,107]. Another benefit of MRI–PET is the reduction of the radiation dose compared with PET–CT, which is crucial in pediatric imaging [98]. Despite the application of MRI–PET in cancer imaging, the evidence for a clear clinical benefit over other well-known methods such as PET–CT is still missing; therefore, additional investigations should be carried out.

#### 4.1.2. MRI–SPECT Dual Imaging for Cancer Diagnosis

MRI–SPECT dual-modality imaging is another type of imaging combination that offers the possibility of obtaining information on molecular processes with anatomical and structural value.

**Figure 13:**
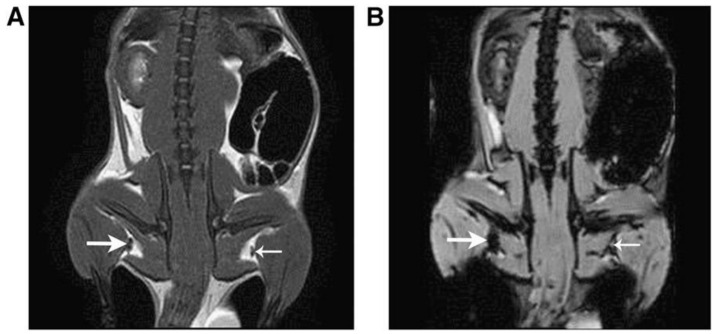
Representative coronal MR images of rats visualizing the sentinel lymph node (SLN) (thick arrow) at the injection side and contralateral node (thin arrow). Accumulation of tracer is clearly visible in the SLN in both a T2-weighted spin-echo (SE) pulse sequence image (**A**) and a gradient-echo (GRE) pulse sequence was used for the T2*-weighted image (**B**). Adapted with permission from [109], Copyright 2011 American Chemical Society.

Madru et al. studied a dual contrast agent based on ^99m^Tc-labeled PEG-coated iron oxide nanoparticles for the imaging of the sentinel lymph node. This lymph node is considered the first to receive the lymphatic drainage from a tumor; therefore, if present, metastases should be imaged there. The MRI–SPECT method was used to identify this lymph node and provide pre-surgical information regarding its characteristics and location. The results demonstrated a clear visualization of the lymph node by SPECT and the uptake of the contrast agent by MRI. This approach could be used for breast cancer and malignant melanoma imaging (Figure 13) [108].

In another approach, Sun et al. used a dual MRI–SPECT modality to characterize the tumor-targeting properties of iron oxide nanoparticles (IONPs) as a contrast agent. For this purpose, IONPs were covalently conjugated with cRGD peptides (which specifically targets integrin αvβ3 receptor overexpressed in tumor cells) and labeled with 125I- nuclide. This probe was administered and it proved to be very helpful for T1/T2 contrast in MRI and SPECT imaging of tumors in vivo, providing higher resolution and sensitivity than both techniques separately [110].

### 4.2. MRI–CT Dual Imaging in Cancer Diagnosis

Computed tomography (CT) is a widely available medical imaging technique based on the varying attenuation of X-rays by tissues within the body, and it needs contrast agents to get good quality images. However, conventional contrast agents such as barium- and iodine-based materials (barium sulphate, iopamidol, and iodixanol) generate some side effects; therefore, recent research has been focused on creating different contrast agents which provide better X-ray attenuation and contrast [101].

Like MRI, CT provides excellent anatomical and morphological results, with high spatial resolution and no tissue penetration limit, although it is relatively weak in functional imaging. Therefore, the components of this dual mode share almost the same functional features. The main differences between them are the physics involved in the respective imaging processes and the contrast agents used for each [11].

**Figure 14:**
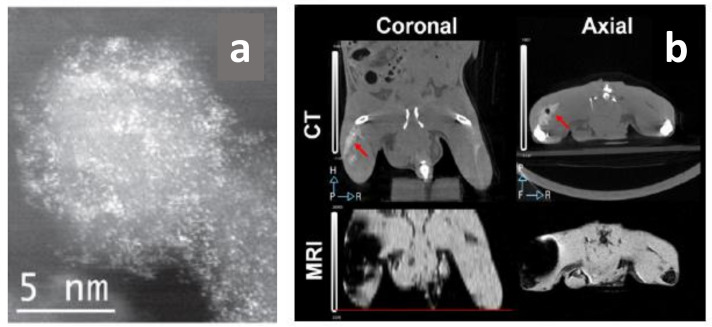
(**a**) STEM HAADF images of FeBi nanocrystals (the bright points correspond to bismuth atoms); (**b**) coronal and axial images taken by CT and MRI after the subcutaneous administration of 100 μL of FeBi@SiPEG (157 mM Fe and 14.6 mM Bi). The location of the contrast in the left leg of the mouse was marked with an arrow in the CT pictures. The black dot is an air bubble accidentally injected. Adapted with permission from [112], Copyright 2015 IOP Publishing Ltd.

To merge both imaging modalities, a mixture of gold or bismuth nanoparticles (CT contrast agents) and SPIONs (MRI contrast agents), establishing a hybrid that can be used as a dual contrast agent for these two modalities, has been described (Figure 14) [111,112]. This hybrid nanosystem is based on an iron oxide core and a gold/bismuth-layered shell and it is able to provide better contrast than iodine agents in CT and also relatively high T2 relaxivity in MRI. According to these results, these contrast agents provided synergistic effects in both techniques (much better X-ray attenuation and contrast), demonstrating that multimodality is superior to a single modal system [101].

## 5. Magnetic Nanoparticle-Based Non-Traditional Multimodal Imaging for Cancer Diagnosis

Besides the conventional imaging applications described above, magnetic nanoparticles (MNPs) can be also applied in emergent multimodal imaging techniques such as magnetic particle imaging (MPI), magneto-motive ultrasound imaging (MMUI), and magneto photoacoustic (MPA) imaging. In combination with traditional imaging modes (e.g., MRI and ultrasound (US) imaging), which provide anatomical information, new imaging modalities can be used to establish the position of magnetic nanoparticles in biological objectives. These novel multimodal imaging techniques that only employ magnetic nanoparticles as tracers improve the sensitivity, resolution, and precision of multimodal imaging abilities [18].

### 5.1. Magnetic Particle Imaging (MPI)

MPI is a new tomographic tracer-based imaging technique, extremely promising in the biomedical field due to its attractive features [113,114,115]. Indeed, it is safe (avoids the use of radiation), quantitative, and fast (resolution of the image in milliseconds). Moreover, it presents excellent spatial resolution (<1 mm) without limits of penetration depth (for the utilization of magnetic fields) and it is background signal-free and has reasonably high sensitivity (200 nM Fe) and specificity. Moreover, it can be used in combination with other image techniques [18,113,116,117,118].

**Figure 15:**
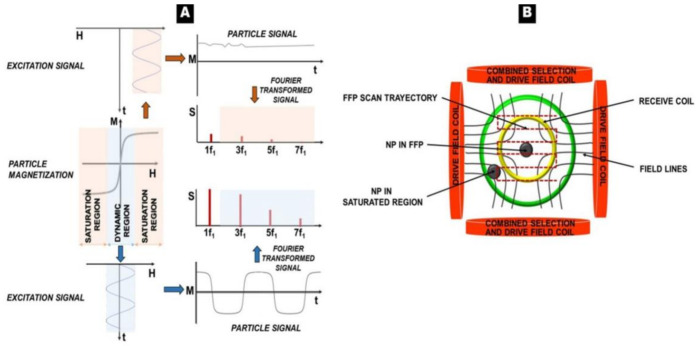
MPI basic concepts. (**A**) Response of SPIONs in dynamic and saturation regions and respective particle and electromagnetic signals; (**B**) scheme of an MPI scanner. Adapted with permission from [15], Copyright 2021 Elsevier Ltd.

The MPI signal is obtained through non-linear magnetization of the magnetic NPs [18]. In the presence of an external magnetic field (H), the NPs’ magnetic moments are disposed parallel to the H direction, creating a magnetization vector (M). The correlation between H and M is nonlinear due to M being saturated when H is too large. When the applied H is oscillating (H^D^(t), drive field), the NPs generate an M that varies with the time (M(t)) and involves electromagnetic signals that are transformed into images (Figure 14). To choose the position of the NPs in their signal, a static H (selection field H^S^ (r)) must also be used. In this manner, the NP signal is exclusive for each NP spatial position [114]. The application of the selection field results in a dynamic and saturation areas. The magnetic NPs inside the dynamic region respond to the drive field shifting their magnetic response, while those inside the saturation zone present the same magnetization values. The centre of the dynamic region shows a field-free point (FFP) or field-free line (FFL) in which the NPs responsible for the MPI signal are located. The entire image is obtained with MPI is by scanning every point of the FFP or FFL in the concerned field of view [18,113,114] (Figure 15). In this way the magnetic NPs can be traced, acquiring a temporal and spatial NP distribution directly proportional to the NP concentration [113,114,116,119].

MPI requires MNPs as tracers to acquire the image and their physical-chemical properties (size, shape, magnetic material, and coating) can affect the sensitivity and resolution of the MPI imaging. The main requirements of MNPs for MPI imaging are: (i) superparamagnetic behavior, (ii) the capacity to be magnetically saturated, and (iii) the ability to exhibit a nonlinear magnetization curve [115]. In general, SPIONs with superior crystallinity features and larger and monodispersed sizes (≥20 nm), presenting higher magnetic moments and high-quality images, make the best MPI tracers [18,115,120,121].

Actually, an ideal tracer for MPI has not yet been developed. Commercial SPIONs such as Resovist^®^ are used as MPI tracer references that provide an adequate signal [18,115,122]. Simulation studies indicate that MNPs with a core size of 30 nm are able to produce a 30-fold improvement in the MPI signal compared to commercial NPs [18,123,124,125,126].

Shape and coating are also two important factors when identifying ideal MPI tracers. The shape mainly affects the crystallinity of the NPs, whereas the coating is key to stabilize and protect the NPs in physiological environments, confer biocompatibility, and guarantee long blood circulation time. MNPs can be encapsulated in cells [121], liposomes [127], or polymers such as dextran [121,128,129] and polyethylene glycol (PEG) [61,130,131,132,133]. The selection of the coating depends on both the final application and biomedical goal [134,135].

MPI can be utilized for imaging and monitoring diseases in several clinical applications, such as for cardiovascular diseases [113,136], cerebrovascular diseases [116,137,138,139], lung perfusion imaging [140], cancer diagnosis [118], and tracking of labeled stem cells [141,142,143]. Additionally, this imaging technology is also helpful in interventional applications [144] in which it can be used as catheter guidance [145]. Considering the scope of this review, below we report some representative examples of MPI applications in oncology. In fact, MPI has been shown to be a promising and extremely useful molecular imaging technique for cancer diagnosis due to its capacity to precisely identify and measure the distribution of MNPs in living targets.

For example, Yu et al. have designed a 3D MPI scanner with which they carried out the first in vivo MPI cancer imaging [117]. They demonstrated the efficacy, high resolution, and safety of this imaging technology. PEG-coated SPIONs were administered intravenously in rats and mice bearing xenograft breast tumors. It was possible to track in vivo the long-circulating SPIONs for 6 days, demonstrating vascular accumulation around the tumoral mass, the EPR effect, and consequent clearance. Thanks to high-resolution images, it was also possible to produce a quantitative evaluation of tracers (Figure 14).

**Figure 16:**
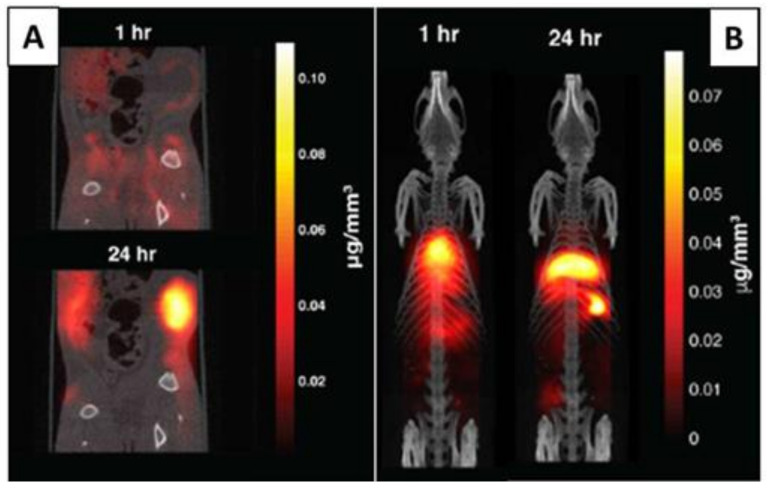
(**A**) Tracer dynamics in group A rats. Slices through the MPI volume over time are co-registered to corresponding CT slices. The MPI images show the SPIONs’ dynamics, from initial rim enhancement and accumulation until the maximum signal. (**B**) Tracer dynamics in group B rats. Maximum intensity projection of 3D MPI volumes co-registered with CT skeletal reference. Adapted with permission from [117], Copyright 2017 American Chemical Society.

Subsequently, additional in vivo studies have been carried out considering the combination of MPI with other imaging techniques [118]. Multimodal cancer imaging using MPI coupled to near infra-red fluorescent (NIRF) imaging, single-photon emission computed tomography (SPECT), and computed tomography (CT) have been used to obtain more accurate monitoring of the tracers’ accumulation in the tumor and biodistribution, obtaining 2D and 3D tomographic images with positive contrast and high resolution (Figure 16).

In another recent study, Mason et al. [146] evaluated the use of MPI for intraoperative margin monitoring in breast-conserving surgery (BCS). They injected iron oxide nanoparticles as a tumor marker and used two types of MNP detectors: a magnetic particle detector to locate residual tumors in the breast and a small MPI scanner to image tumor distribution in the excised specimen. With this study they demonstrated that MPI offers high sensitivity with short acquisition times and can be used during surgery to obtain a complete removal of the tumor and thus a better prognosis of the disease. If it were possible to combine these results with another imaging modality, the distance between the tumor and the surface could be determined, thus providing crucial information for the operation.

**Figure 17:**
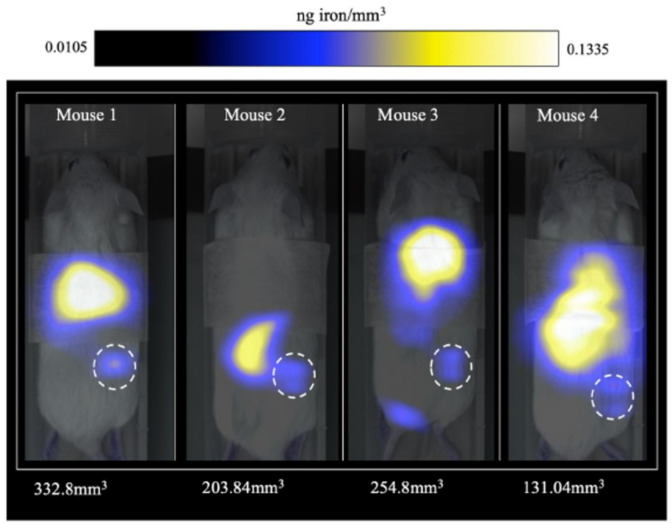
MPI signal representing iron-labeled experimental CTCs detected in the lower right mammary fat pad (MFP) tumors of all mice. Adapted with permission from [148], Copyright 2021 The Royal Society of Chemistry.

More recently, tumor-associated macrophages (TAM) have been labeled with commercial SPIONs and visualized by MPI and MRI for comparison [147]. The MPI provided quantitative information about SPION-labeled macrophage distribution, as well as higher sensitivity and specificity compared to MRI. For the first time, the utilization of MPI for the identification of administrated iron-labeled circulating tumor cells (CTCs) and the examination of tumor self-homing in mice with human breast cancer was also verified (Figure 17) [148].

Interestingly, MPI is becoming a tomographic imaging technology with excellent abilities for clinical use. However, some obstacles, such as the simultaneous optimization of hardware, image reconstruction algorithms, and tracers, must be overcome before complete medical clinic translation is possible. The main disadvantage of MPI is the lack of anatomical information and it is therefore preferably combined with either CT or MRI; indeed, industrial efforts are underway to build an MPI/MRI scanner. Furthermore, another relevant hurdle in the clinical deployment of MPI is represented by the obtainment of an optimized MPI tracer, a crucial factor to improve imaging sensitivity and in vivo tracking [114,122,149].

### 5.2. MNP-Assisted Multimodal Ultrasound Imaging

Ultrasound (US) imaging is extensively used for the detection of metastatic tumors, such as in echocardiography and angiography. This technique can acquire real-time imaging, high resolution, and good penetration depths and can be also portable. Nevertheless, its sensitivity abilities are limited. Recently, it has been reported that US sensitivity can be enhanced by using MNPs, thus enabling MNP-assisted multimodal US imaging as an effective technique for real-time visualization at the cellular and molecular levels. There are two types of representative multimodal US imaging: magneto-motive ultrasound imaging (MMUI) and magneto photoacoustic (MPA) imaging [18].

#### 5.2.1. Magneto-Motive Ultrasound Imaging (MMUI)

**Figure 18:**
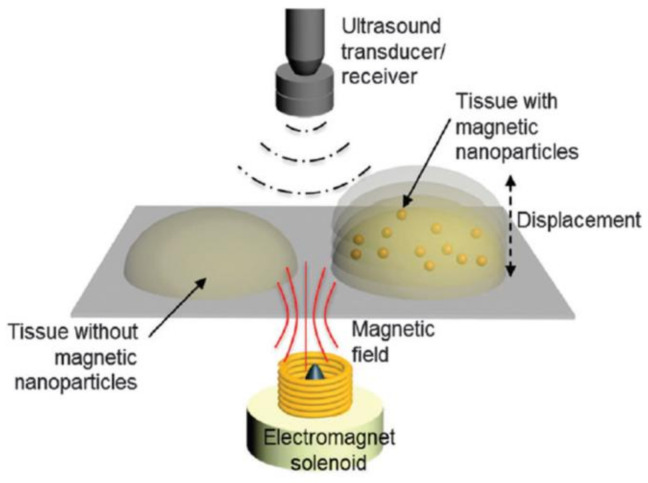
Schematic overview of a magneto-motive ultrasound imaging (MMUI) system. Magnetically induced displacement of the tissue embedded with magnetic nanoparticles generates an MMUI signal, which is detected using an ultrasound receiver. Adapted with permission from [18], Copyright 2015 The Royal Society of Chemistry.

In the MMUI technique, MNPs are exposed to a pulsated magnetic field characterized by elevated intensity, producing a displacemente (movement) that can be observed using US imaging. During their magnetically stimulated displacement (magneto-motive force), MNPs suffer a resistence presented by the biological tissue (tissue resistence force). Due to these two forces, MNPs produce vibrations which are dected by ultrasound (US)-based motion-monitoring imaging [18]. Thus, US signals locate MNPs in the biological tissues thanks to microvibrations generated by the interations of these NPs in the presence of an external magnetic field [150]. MNPs are capable of responding to the magnetic field due to their magnetic susceptibility (χ), which implies that the MMUI signal is direcly proportional to the MNPs’ χ. As a consequence, the higher the χ of the MNPs is, the more accurate the imaging technique will be (Figure 18) [18].

MMUI technology has proved to have huge potential as a novel and safe method for imaging at the cellular and molecular levels, such as in sentinel lymph node (SLN) detection, but also for stem cells and drug delivery tracking. Moreover, it can be applied for the detection of tissue defects and/or regeneration due to the magnetically induced movement of the SPIONs in the tissue varying with its viscoelastic characteristics, associated with biological environments [18].

**Figure 19:**
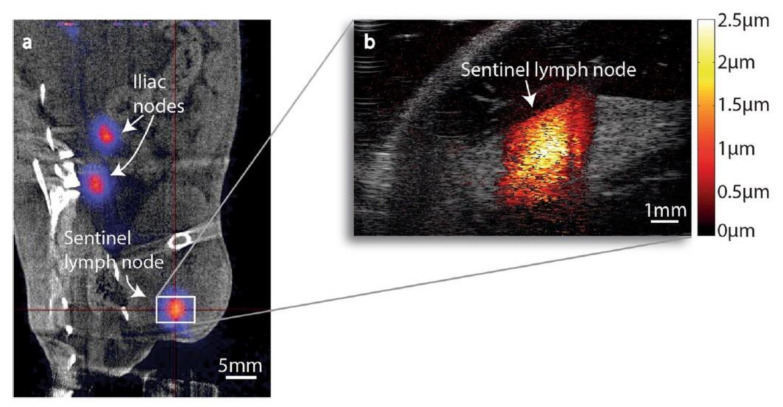
(**a**) Coronal PET/CT image of animal 1. The centre of the red cross shows the position of the nanoparticle-accumulated sentinel lymph node. Nanoparticle accumulation can also be seen in two iliac lymph nodes. (**b**) MMUI image of the same sentinel lymph node. Figure adapted from [152], Copyright 2017 Nature Research.

In certain kinds of cancer, such as colon rectal cancer, it is essential to detect the sentinel lymph node to confirm the efficacy of the oncological treatment in achieving a targeted surgical resetion with enhanced life quality for the patient [151]. To identify metastases during the surgery, intra-surgical guidance can currently be obtained by employing radioactive tracers [152]. MMUI is a safe multimodal imaging technique that uses SPIONs as contrast agents and presents the possibily of distinguishing the sentinel lymph node (Figure 19) [18,151,152].

However, to reach clinical translation, some technological improvements are required, such as in the design (e.g., position, size, etc.) of the external magnet and the engineering of the NPs, as well as enhancement of the excitation and signal processing. Current experiments are improving real-time performance in MMUI imaging. This makes MMUI a robust candidate for ultrasonic molecular imaging, as it would prevent the use of ionizing radiation while showing similar spatial resolution and sensitivity [153].

#### 5.2.2. Magneto Photoacoustic Imaging (MPA)

MPA is a new and attractive noninvasive technique for molecular imaging of cells and tissues that is attracting great attention for the detection of tumor-circulating cells [154,155,156]. A great advantage of MPA is its high sensitivity due to the suppression of the tissue background signal. Other advantages of this technique are that it makes it possible to differentiate between damaged and healthy tissue with high image resolution and deep penetration.

**Figure 20:**
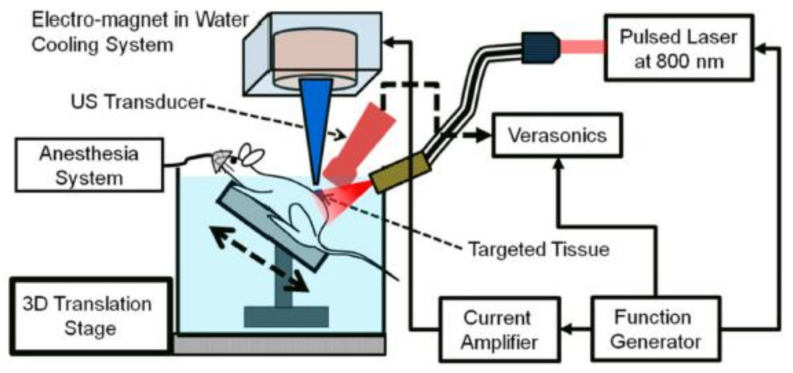
Schematic of the in vivo magneto-photo-acoustic imaging system. Adapted with permission from [158], Copyright 2015, American Chemical Society.

In photoacoustic (PA) imaging the laser light absorption produces the thermo-elastic expansion of the tissue which in turn generates the US waves. The US waves are detected and converted in electric signals that are processed as PA images (Figure 20). As PA and US have very similar instrumentation, they can be used together (PA–US dual-modal imaging): PA can provide high-contrast images while US gives anatomical and morphological details. A disadvantage of this multimodal imaging system is associated with the PA background signals generated from endogenous photo-absorbers present in the tissue [18]. The use of MNPs, resulting in magneto-PA (MPA), can improve the efficacy of this method [157] because the only vibrations produced are by the MNPs when an external magnetic field is applied. It is possible to consider MPA as a synergistic combination of PA with the MMUI technique. As contrast agents, magneto-optical NPs are used; these are NPs with a magnetic core and gold shell [157,158].

**Figure 21:**
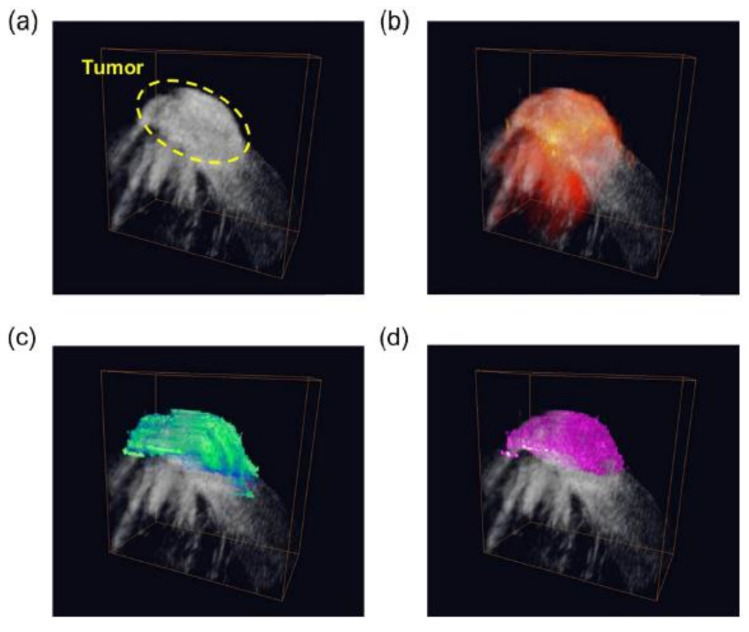
3D (**a**) ultrasound, (**b**) photoacoustic, (**c**) magneto-motive ultrasound, and (**d**) magneto-photo-acoustic images of the LNP accumulated in the tumor area and the background tissue in a mouse. Figure adapted from [156], Copyright 2014 Elsevier GmbH.

MPA is principally used to detect tumor-associated macrophages (TAM). In fact, the ability of this method to noninvasively detect NP delivery to the tissues, such as their injection into the cells, has been demonstrated [155]. MPA can be considered a combination of PA and MMUI, and both methods can detect MNPs, so their signals intesify with increases in the number of NPs; this is when the NPs are delivered to the tissue. When the magnetic contrast agents are internalized into macrophages by endocytosis, the PA signal should not change a lot because the iron concentration does not change. In contrast, when NPs internalized inside the cells create magnetic aggregates, this makes the MMUIS signal increase. The MPA signal should increase when NPs are internalized into the macrophages and it is obtained by normalization of the MMUI signal by the PA signal. In other example, SPIONs and gold nanorods encapsulated in liposomes, possessing optical absorption and, at the same time, magnetic properties, were administrated in vivo in mice with xenograft tumors (Figure 21) [156]. The PA signal was produced from liposomes and from endogenous absorbers already present in the tissue. However, the PA imaging contrast was increased by the magnetic response of liposomial NPs and it was able to suppress the unwanted tissue background. In another study, Arnal et al. [159] presented a new magneto-optically coupled core-shell nanoparticle composed of an iron oxide nanoparticle the surface of which had been copolymerized with polypyrrole. With this approach, they solved the photo-instability and the small-scale production problems and extended the optical absortion coefficient.

Nonetheless, despite its great potential, the development of MPA is only at its beginning and more studies must be performed for its translation to clinic applications.

## 6. General Remarks and Future Perspectives

As clearly described within this review, over the last two decades the field of nanotechnology has experienced tremendous growth and advancement, and its application in molecular imaging research has resulted in the development of a wide set of imaging agents based on inorganic nanostructures and functionalized with a various range of ligands. Due to the flexible nature of magnetic nanoparticles and the related synthetic progresses, the expansion of specific custom-made probes with unique properties, such as superior targeting abilities and enhanced in vivo half-lives, is now a tangible reality. Due to these features, numerous multimodal imaging contrast agents based on magnetic nanoparticles have been established as potent tools for accurate cancer diagnosis using complementary combinations of more than one contrast agent. In fact, on the one hand, thanks to the fine tuning of the synthetic methodological design, it is possible to control the size, composition, and shape of the nanoparticles, thus modifying their magnetism in a controlled way and enhancing NMR contrast effects, which finally results in more sensitive multifunctional nanosystems. On the other hand, thanks to functionalization with other imaging moieties (e.g., fluorescent molecules and radioisotopes), nanoparticles with multimodal imaging capabilities can be achieved. Interestingly, these multimodal imaging probes not only provide the means for complementary imaging of the same region of interest but can also enable the imaging of different regions with individual imaging techniques. As a result, more comprehensive and reliable diagnosis is possible with smaller numbers of nanoparticle probes than with each individual imaging modality.

These magnetic nanoparticle-based multimodal imaging techniques have great potential to secure better imaging sensitivity and accuracy for better knowledge of biological systems and precise imaging of biological targets. As an overall result, cancer diagnosis has improved notably, thus enabling the shortening of detection times and allowing tumoral disease imaging at the subclinical level.

Nevertheless, nanomaterials have intrinsic problematic factors in comparison to small-molecule drugs that still have to be understood and analyzed through research. In fact, while numerous topics regarding the biodistribution and clearance of nanoparticles in vivo have been investigated over the past decade, our current knowledge is still far from complete.

For example, the biosafety of inorganic nanoparticle agents is still a crucial unsolved problem, and it should be evaluated carefully to fully draw out the potential of nanoparticle agents in molecular imaging. Therefore, although nanoparticles have shown solid results as multimodal molecular imaging agents, proper safety assessments regarding their toxicity are absolutely necessary before their ultimate use in the clinic. Actually, this systematic toxicological study is poised to be one of the most important steps to taken by nanomedicine toward its real and definitive clinical translation.

Furthermore, some relevant techno-economic challenges must be still addressed in order to enable the definitive clinical application of these hybrid multifunctional nanoparticles. On the one hand, the development of the engineering of multimodal imaging equipment (e.g., PET–MRI) must be improved, as it is still extremely expensive and requires complex infrastructures, with applicability being unfeasible for most of the national health systems. Therefore, technological solutions making these multimodal devices lighter, cost-effective, and more adaptable to the applications need to be researched further. On the other hand, achieving high reproducibility in the production of contrast agents based on these multifunctional hybrid nanoparticles is mandatory in order to permit their industrial scale-up. Once the reproducibility requirement has been achieved, the multifunctional nanoparticles that present greater sensitivity with lower production costs can be selected [26]. Fortunately, the research in these directions is already ongoing and many promising solutions have already been proposed [160].

In addition to the latter, other research lines worth mentioning are under development with the aim of solving the existing issues and improving the overall strategy described in this review. For example, it has been demonstrated that the combination of augmented reality techniques with multimodal image guidance facilitates surgical planning, intraoperative imaging, and treatment monitoring. With this strategy, surgeons can be provided with more precise complementary information of structural imaging and functional imaging, therefore easing surgery development and improving patient prognosis [161]. Another interesting future research line is related to the application of artificial intelligence (AI) in molecular imaging [162]. In fact, AI in general and deep learning in particular have, to varying degrees, impacted almost all aspects of molecular imaging, from image acquisition to diagnosis, especially in imaging processing. As a result, great precision in the diagnosis and follow-up of cancer patients has been achieved [162].

In conclusion, although most of the advances presented herein have yet to be definitively adopted in the clinic, the rapid pace of discoveries in this field augurs well for increased robustness, transparency, and trustworthiness in the use of hybrid magnetic nanoparticles for the multimodal molecular diagnosis of cancer, which will ultimately lead to improved patient care.

## Figures and Tables

**Table 1:** Pros and cons of individual and combined imaging modalities used in molecular imaging and discussed within this review.

Imaging Modality	Information	Advantages	Disadvantages
MRI	Anatomical	High spatial resolution, no tissue penetration limit	Low sensitivity, long imaging time
Optical imaging	Molecular	High sensitivity, fast acquisition, low cost	Poor spatial resolution, small penetration depth
CT	Anatomical	High spatial resolution, strong penetration depth, fast acquisition	Radiation risk
PET	Molecular	High sensitivity, strong penetration depth	Poor spatial resolution, radiation risk
SPECT	Molecular	High sensitivity, strong penetration depth	Poor spatial resolution, radiation risk
US	Anatomical	High sensitivity, low cost, fast acquisition	Poor spatial resolution
MRI–optical imaging	Anatomical/molecular	High sensitivity, high spatial resolution, no tissue penetration limit	High cost
MRI–CT	Anatomical	High spatial resolution, no tissue penetration limit	Radiation risk, high cost, low sensitivity
MRI–PET/SPECT	Anatomical/molecular	High spatial resolution, high sensitivity, no tissue penetration limit	Radiation risk, high cost
MRI–US	Anatomical	High spatial resolution, no tissue penetration limit, high sensitivity	High cost

**Table 2:** Examples of the major methods of synthesizing magneto-fluorescent hybrid nanosystems.

Association			
Nanoparticles	Optical Agent	Application	Reference
Iron oxide	Quantum dot	Cervical cancer	[77]
Iron oxide	Quantum dot	Cervical cancer and neural cells	[75]
Iron oxide	Carbon dots	Cervical cancer	[76]
Silica with iron oxide nanoparticles	Rhodamine dye	Neuroblastoma	[78]
**Encapsulation**			
**Nanoparticles**	**Optical Agent**	**Application**	**Reference**
Silica with iron oxide nanoparticles	Rhodamine dye	Neuroblastoma	[78]
Mesoporous silica nanoparticles (MSNs) with Gd-DTPA	Heptamethine dye (IR-808)	Glioblastoma	[79]
Silica with iron oxide nanoparticles	Quantum dots	Mammary carcinoma	[80]
**Dispersion**			
**Nanoparticles**	**Optical Agent**	**Application**	**Reference**
Copolyarylene ether nitriles with iron oxide nanoparticles	Quantum dots	Mammary carcinoma	[81]
Ethylene oxide polymer vesicles and iron oxide nanoparticles	(7-Diethylamino coumarin)-3-carboxyic acid (DEAC-CA)	Cervical cancer	[82]
Polymeric micelles and MnFe_2_O_4_ magnetic nanoparticles	Quantum dots	Glioblastoma	[83]
Polymeric matrix with iron oxide nanoparticles	Chlorin e6 dye (Ce6)	Colon cancer	[84]
Liposome with iron oxide nanoparticles	Texas Red dye	Ovarian cancer	[85]

## Data Availability

The data presented in this study are available on request from the corresponding author.
